# *In Vitro*-Stimulated IL-6 Monocyte Secretion and *In Vivo* Peripheral Blood T Lymphocyte Activation Uniquely Predicted 15-Year Survival in Patients with Head and Neck Squamous Cell Carcinoma

**DOI:** 10.1371/journal.pone.0129724

**Published:** 2015-06-16

**Authors:** Helene Hersvik Aarstad, Olav Karsten Vintermyr, Elling Ulvestad, Kenneth Kross, John Helge Heimdal, Hans Jorgen Aarstad

**Affiliations:** 1 Department of Clinical Medicine, Faculty of Medicine and Dentistry, University of Bergen, Bergen, Norway; 2 Department of Otolaryngology/Head and Neck Surgery, Haukeland University Hospital, Bergen, Norway; 3 Department of Pathology, Haukeland University Hospital, Bergen, Norway; 4 Department of Microbiology, Haukeland University Hospital, Bergen, Norway; 5 Department of Clinical Science, Faculty of Medicine and Dentistry, University of Bergen, Bergen, Norway; 6 Department of Otolaryngology/Head and Neck Surgery, Maastricht University Medical Centre, Maastricht, The Netherlands; University of Oslo, NORWAY

## Abstract

The study was performed in order to determine whether peripheral blood monocyte *in vitro* function, and lymphocyte *in vivo* activation at diagnosis, was associated with HPV tumor infection status and 15-year survival in head and neck squamous cell carcinoma (HNSCC) patients. Sixty-five patients from a consecutive cohort of newly diagnosed HNSCCs, together with 18 control patients, were included in the study. Monocyte responsiveness was assessed by measuring monocyte *in vitro* interleukin (IL)-6 secretions after 24 hours of LPS stimulation in cultures with a serum-free medium. T lymphocyte activation was determined as the fraction of CD71-positive cells on CD3-positive cells by flow cytometry, whereas HPV infection was determined by PCR on formalin-fixed paraffin-embedded (FFPE) tumor tissue. Disease-specific survivals and overall survivals were determined 15 years following inclusion. HPV-positive HNSCC patients had a lower monocyte LPS-stimulated IL-6 response. A high LPS-stimulated monocyte IL-6 response predicted a decreased survival rate (*P*=0.019). A high percentage of CD71-positive T lymphocytes also predicted an impaired prognosis (*P*=0.021). The predictive power of IL-6 monocyte LPS-stimulated responses was retained when adjusted for age, gender and TNM stage of the patients. The monocyte and T lymphocyte survival predictions were independent of each other. The survival was particularly low with a combined high activated monocyte and T lymphocyte status. In a multivariate analysis, IL-6 secretion and the percentage of CD71-positive T lymphocytes both uniquely predicted survival independent of HPV infection status. It is postulated that the natural and adaptive immune systems are separately and additionally linked to the clinical aggressiveness of HNSCCs.

## Introduction

Both epidemiological and experimental evidence suggest a link between chronic inflammation and the development of malignancy [1, 2], in particular when the inflammation is caused by chronic infection [3]. Chronic inflammation has also been suggested to promote head and neck squamous cell carcinoma (HNSCC) [4]. Furthermore, the steadily growing knowledge about the human papilloma virus (HPV) as a pathogen of some HNSCC tumors [5], especially the oropharyngeal (OP)SCC [6, 7], should encourage the addressing of inflammation in the development of HNSCC.

The immune system probably takes part in the eradication of HPV-positive (+) tumor cells during treatment [8]. The role of the immune system in cases where HNSCC disease is caused by HPV infection beyond this finding is not well established. Moreover, the potentially different roles of the immune system in HPV-negative (-) versus HPV-positive HNSCC patients have not been established and are therefore of interest to study.

The influx of inflammatory cells into tumors generally indicates a more aggressive clinical behavior [1]. In the oral cavity, ordinary HNSCCs may serve as examples of the latter phenomenon [9]. On the other hand, possibly due to their association with HPV infection, the local inflammatory tumor response has long been associated with a better prognosis in OPSCC patients [10].

The immune system functions as a whole body-integrated system in response to various types of agents, possibly including tumor cells. In HNSCC patients, immune cells from peripheral blood (PB) have, e.g. been shown to be generally responsive to local tumors [4, 11]. Thus, HNSCCs should be considered as affecting the host immune competent cells. If there are discrepancies in HPV+ and HPV- HNSCC patients with respect to the PB immune responses have not been extensively studied and warrant further investigations.

High soluble IL-2α receptor levels in serum may predict impaired prognosis in HNSCC patients [12]. In line with this, we have shown that a high percentage of the PB T lymphocytes carrying the activation epitope CD69 is associated with an impaired long-term prognosis [13]. If this is also the case for other T lymphocyte activation epitopes, such as CD71 [14], both with and without reference to the HPV tumor status is not known and has been one of the specific aims of the present study.

The mononuclear phagocyte (MNP) system, including monocytes, macrophages and some dendritic cells [15], constitutes a major part of the inflammatory system, both as effector cells and by the secretion of various cytokines, such as pro-inflammatory cytokines (IL-6) and chemokines (MCP-1) [4]. Serum IL-6 levels may predict survival in cancer patients [16], including both lung cancer [17] and HNSCC [18] patients. In accordance with this, we have shown that a high IL-6 secretion measured from lipopolysaccharide (LPS)-stimulated PB monocytes from HNSCC patients predicted an impaired intermediate-term prognosis [19]. It therefore seems that increased IL-6 levels are a negative prognostic factor in HNSCC patients, although to what extent this is affected by the HPV infection in the tumor is not known. To help address this issue in this study, we have evaluated the prognostic power of the present monocyte priming by studying both LPS-stimulated IL-6 secretion *in vitro* and these findings by the HPV infection status.

We have studied the prognosis of newly diagnosed HNSCC patients dependent on the spontaneous activation of T lymphocytes *in vivo*, and the level of monocyte priming *in vitro* from the same patients. Both monocyte and lymphocyte functional levels uniquely predicted HNSCC prognosis both with and without HPV stratification.

## Materials and Methods

### Patients

The study comprised consecutive patients hospitalized at the Department of Otolaryngology and Head and Neck Surgery, Haukeland University Hospital, Bergen, Norway. The patients either had squamous cell carcinoma (SCC) (N = 65) or non-cancer diseases of the head and neck (HN) (N = 18), diagnosed in the period from June 1, 1997 to April 12, 1999. Patients with autoimmune disease or patients on corticosteroid medications were not included in the study. The study was approved by the “Regional Committees for Medical and Health Research Ethics,” Western Norway branch, and each patient gave their written consent before participating in the study. The primary sites of the carcinomas were: oral cavity (n = 26), oropharynx (n = 19), hypopharynx (n = 5), larynx (n = 13), maxilla (n = 1) and unknown primary (n = 1). The mean ± standard deviation (SD) ages of the HNSCC patients were 62.1±10.7 years and 64.4±10.6 years for the control patients. The TNM stages of the HNSCC patients are shown in Table 1. The survival of the patients was determined from the Norwegian Population Registry by a survival time of 15 years. Still, 16 of 65 cancer patients and 10 of 18 controls were alive.

**Table 1:** TNM stages of all included patients.

		T stage					
		0	1	2	3	4	SUM
N stage	0	2	10	12	5	8	37
	1	0	0	2	2	3	7
	2	0	6	4	4	3	17
	3	0	3	1	0	0	4
	SUM	2	19	19	11	14	**65**

All patients were M0 at inclusion.

### Blood samples

The patients were included in the study at their arrival to the department before any specific cancer treatment had started. All blood samples were drawn at 7.30 a.m. as a bedside procedure, and each patient was asked to stay in bed until the blood sample was drawn.

#### Monocyte preparation

Peripheral blood mononuclear cells (PBMC) were separated by gradient centrifugation with Lymphoprep (Nycomed, Oslo, Norway) as the density gradient medium. The PBMC yield of 8.5 ml blood was allocated to a 24-well plate (Nunc A/S, Roskilde, Denmark) with RPMI-1640 (BioWhittaker, Walkersville, MD, USA), supplemented with amphotericin B (2.5 μg/ml) and glucose (both Sigma, St. Louis, MO, USA), HEPES, L-glutamine (2 mM), penicillin (100 IU/ml), streptomycin (100 μg/ml), sodium bicarbonate, sodium pyruvate (all from BioWhittaker) and 20% autologous serum (AS) to a total volume of 0.5 ml/well. After 40 minutes of pre-incubation, the adherent monocytes were purified by washing and then cultured in a serum-free medium (UltraCulture; BioWhittaker) with 0.5 ml/well. The method yields more than 95% monocyte-positive cells by non-specific esterase stain with more than 95% viable cells, as tested by trypan blue stain. *In vitro* stimulation was provided for 24 hours by 1 μg/ml lipopolysaccharide (LPS) derived from *Escherichia coli* (Sigma) before the sample collection. Moreover, cultures without specific stimulation were used as background controls.

#### IL-6 analysis

The contents of IL-6 in the supernatants were determined through the use of an enzyme-linked immuno-sorbent assay (ELISA) kit, manufactured by R&D Systems (R&D Systems Europe Ltd., Abingdon, Great Britain). All procedures were performed according to the specifications from the manufacturer. Briefly, 96-well micro-tither plates (Costar Corning, NY, USA) were coated overnight at room temperature (RT) with monoclonal mouse α-human IL-6 capture antibodies. Diluted samples and recombinant human IL-6 standards were added and incubated for 2 hours at RT, followed by the addition of biotinylated polyclonal goat α-human IL-6. The plates were incubated for 20 minutes at RT with streptavidin-conjugated horseradish peroxidase. Tetra-methyl-benzidine (TMB) (Sigma) and H_2_O_2_ were also used as substrates. Absorbency values were measured at 450 nm using Softmax Pro version 4.0 on an Emax Precision micro-tither plate reader (Molecular Devices, Sunnyvale, CA, USA). The lower detection level was 9 pg/mL for IL-6.

#### Flow cytometric determination of percentage positive PBMC cells

Immunophenotyping was performed on each PBMC specimen using a panel of mAbs conjugated with either fluorescein isothiocyanate (FITC) or phycoerythrin (PE) fluorochromes. Anti-CD71-FITC (anti-transferrin receptor) and anti-CD3-PE were obtained from Becton Dickinson, San Jose, CA, USA. Samples with fluorochrome-conjugated nonspecific isotype-matched mAbs were used as negative controls. The cell analysis was performed on a Coulter Epics XL flow cytometer (Coulter Electronics, Ltd, Luton, Great Britain) equipped with an air-cooled 15 mW argon-ion laser operating at 488 nm. In each cell preparation, gates were set on the lymphocytes using light scatter characteristics and 5,000 cells were analyzed. The fluorescence data were expressed as dual parameter histograms of FITC versus PE fluorescence. Moreover, fluorochrome compensation was adjusted utilizing normal control peripheral blood leukocytes labeled with FITC-coupled anti-CD4 and PE-coupled anti-CD8. Four-quadrant analyses, with markers set on the isotype controls, were further used to determine the percentage of positive cells for each set of mAbs.

#### DNA isolation

DNA was extracted from formalin-fixed paraffin-embedded (FFPE) specimens. These were tissues from primary HN tumors in diagnostic or surgical samples, or from lymph node metastatic lesions. Three 10 μm-thick FFPE sections were deparaffinized in xylene and ethanol, and digested overnight in an ATL buffer and Proteinase K (Qiagen GmbH, Hilden, Germany) at 56°C. DNA was extracted using the EZNA tissue DNA kit (Omega Bio-Tek, Norcross, GA, USA). The DNA concentration was measured on a NanoDrop spectrophotometer (Nanodrop, Minneapolis, MN, USA). HPV-negative DNA (control) samples were isolated from blocks provided from human large bowel FFPE tissue samples. For every fourth-sectioned ENT specimen, a control (HPV-negative) block was sectioned as internal negative controls to test for the potential risk of HPV contamination in the procedure. All interspersed negative sample controls had to be negative to accept any positive HPV PCR results.

All tumor samples were reviewed by an expert in pathology (OKV), and representative FFPE tissue samples were selected for HPV DNA analysis.

#### HPV DNA detection

We have previously published our method in detail [20]. For the detection of HPV, DNA standard Gp5+/Gp6+ primers were used [21]. The PCR reaction mix consisted of a Multiplex Mastermix PCR kit (Qiagen GmbH, Hilden, Germany), 2 μl of primer mix and 2 μl of DNA run in a volume of 20 μL. The samples were inactivated by heating (95°C) for 15 minutes and then run for 38 cycles at 94°C for 45 seconds, 43°C for 90 seconds and 72°C for 90 seconds, and lastly 72°C for 10 minutes. Samples with known HPV DNA were included as positive (PCR) controls, whereas the substrate DNA was omitted in the negative (PCR) controls.

The PCR products were separated and visualized on a 3% agarose gel. Only samples with distinct PCR bands were considered positive for HPV and further processed for HPV subtype identity by DNA sequencing. The PCR products were first purified by incubation with ExoStar (GE Healthcare, Buckinghamshire, UK) at 37°C for 15 minutes, followed by enzyme inactivation at 80°C for 15 minutes. The purified PCR products were then prepared for sequencing using the same primers as for the initial PCR reaction in combination with a BigDye Terminator v1.1 Cycle Sequencing kit (Life Technologies, Foster City, CA, USA). Before analysis on a 3130XL Genetic Analyzer (Life Technologies, Foster City, CA, USA), the products of the sequencing reaction was purified using a BigDye Xterminator kit (Life Technologies, Foster City, CA, USA). The HPV DNA sequences were identified using the NCBI BLAST database, and more than a 98% homology was observed with NCBI BLAST.

#### HPV status of included patients

Ten HNSCC patients from the original cohort were positive for HPV DNA, all with tumors originating from oropharynx [13]. These tumors originated from the tonsils (n = 7), the basis of the tongue (n = 2) or the lateral wall of the oropharynx (n = 1). We lacked the actual biopsies from three HNSCC patients of the present series in order to determine HPV status. None of these patients had tumors originating from the oropharynx, so we have therefore designated them as HPV- patients during the following reported analyses.

### Statistical analysis

The statistical program package IBM SPSS Statistics for Windows was employed (Rel.19.0.0. 2009. SPSS Inc.). Numbers are given as mean ± SD. The groups were compared by Chi square tests, while survival analyses were performed by Cox regression or Kaplan-Meier analyses. In some analyses, HNSCC patient monocyte responsiveness and T lymphocyte activation levels were dichotomized as high or low responders by the median value. Statistical significance was considered if *P*<0.05.

## Results

### HNSCC patient prognosis by HPV infection status

**Fig 1:**
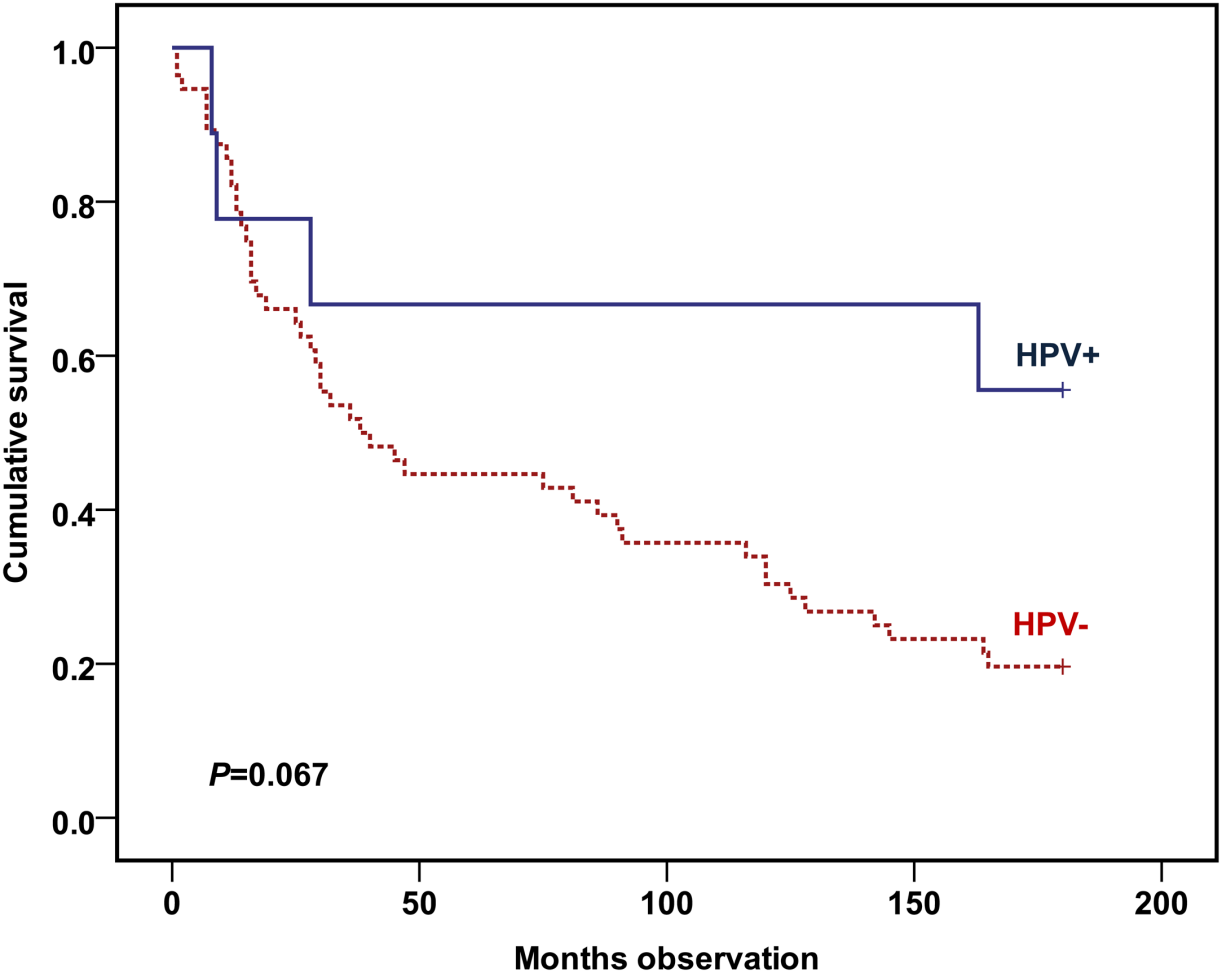
Overall survivals of the cohort of included HNSCC patients stratified by whether there were HPV-positive or HPV-negative tumors. Cohort: 56 HPV-negative and 9 HPV-positive HNSCC patients. Statistics by Kaplan-Meier analysis (Log rank test).

A strong trend was shown towards better survival among the HPV+ patients (*P* = 0.067) (Fig 1).

### Monocyte IL-6 secretion in control versus HNSCC patients

Analyses showed that the supernatant IL-6 levels in cultures from control versus HNSCC patients were not statistically different, both as measured with and without LPS stimulation. Similar results were obtained when the background IL-6 levels were subtracted (Table 2). No difference was observed as to the percentage of CD71-positive T lymphocytes between the control and HNSCC patients (Table 2).

**Table 2:** HNSCC or control patient PB CD71 positivity (%) on T lymphocytes (CD3-positive cells) or monocyte 24 hours *in vitro* LPS-stimulated IL-6 secretion (pg/ml).

	Control			HNSCC			p-value
	Mean	SD	Range	Mean	SD	Range	
	Monocyte spontaneous *in vitro* 24-hour cytokine secretion						
**IL-6**	8210	13039	1–44747	3389	6409	0–26463	n.s.
	Monocyte LPS-stimulated *in vitro* 24-hour cytokine secretion						
**IL-6**	51794	39837	7135–131011	51347	35653	1–139794	n.s.
	Monocyte LPS-stimulated *in vitro* 24-hour net cytokine secretion						
**IL-6**	43588	34702	6857–127575	47835	33865	0–133014	n.s.
	PB T lymphocytes CD71+ (%)						
**CD71/CD3**	8.3	4.6	1.0–8.3	9.2	6.0	1.6–25.4	n.s.

### Monocyte IL-6 secretion in HNSCC HPV+ versus HNSCC HPV- patients

Analyses showed that the supernatant IL-6 levels in cultures from HNSCC HPV- patients (55333±34550 pg/ml) were higher compared to the HNSCC HPV+ (24020±33039 pg/ml) patients (*P* = 0.002). Similar results were obtained when the background IL-6 levels were subtracted (Table 3).

**Table 3:** HPV+ HNSCC or HPV- HNSCC patient PB T lymphocyte (CD3+) CD71+ (%) or monocyte 24 hours *in vitro* LPS-stimulated IL-6 secretion (pg/ml).

	HNSCC						
	HPV-			HPV+			p-value
	Mean	SD	Range	Mean	SD	Range	
	Monocyte spontaneous *in vitro* 24-hour cytokine secretion						
**IL-6**	3582	6732	0–26463	2011	3280	1–9286	n.s.
	Monocyte LPS-stimulated *in vitro* 24-hour cytokine secretion						
**IL-6**	55333	34550	8117–139794	24020	33039	1–93947	.002
	Monocyte LPS-stimulated *in vitro* 24-hour net cytokine secretion						
**IL-6**	51602	33003	275–133014	22008	29962	0–84661	.007
	PB T lymphocytes CD71+ (%)						
**CD71/CD3**	9.6	6.2	1.6–25.4	7.0	4.2	1.6–14.5	n.s.

### Prognostic value of monocyte *in vitro* activation

**Fig 2:**
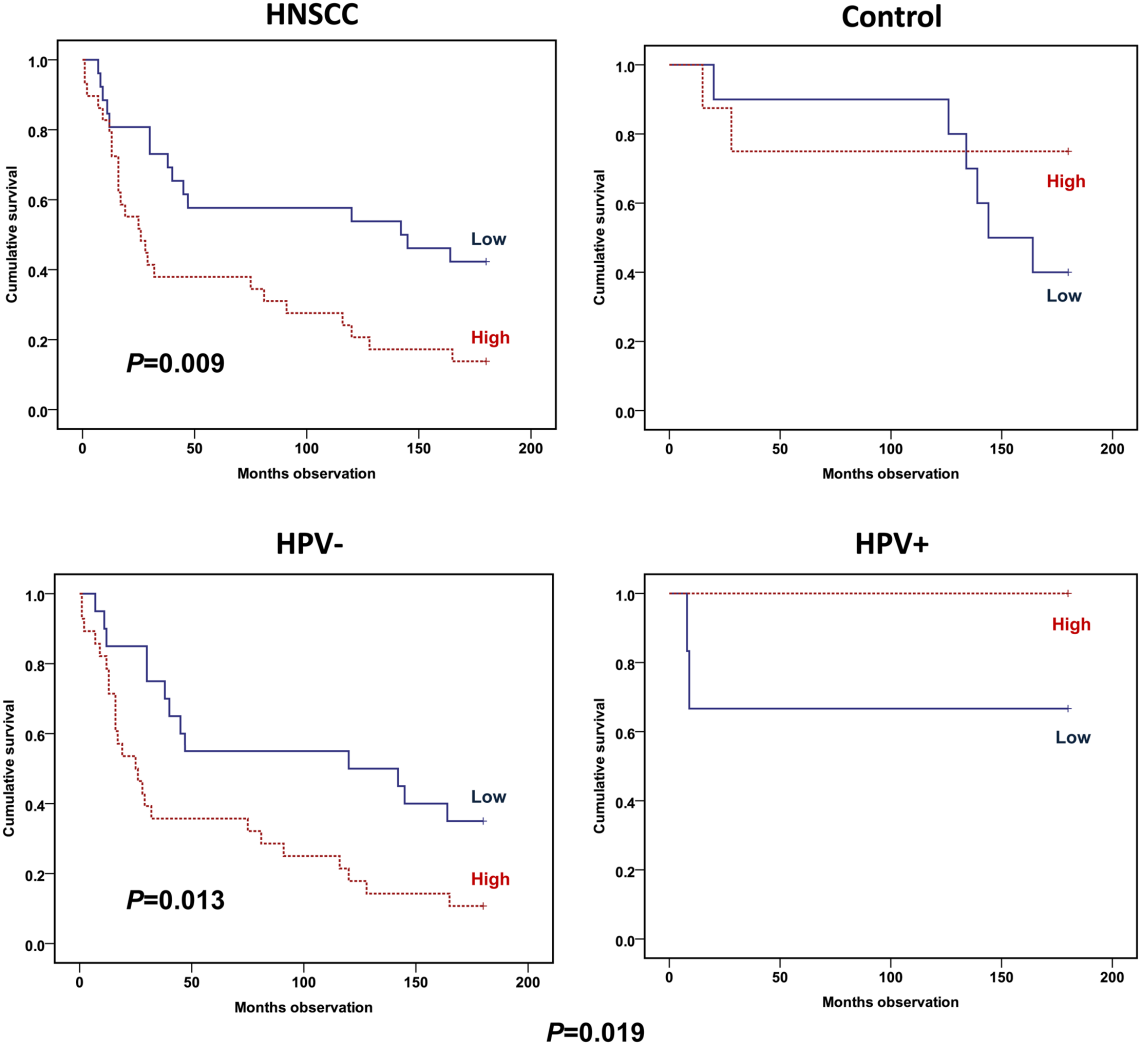
Overall survival plots by IL-6 *in vitro* secretion from LPS-stimulated monocytes. HNSCC versus control patients (upper panel) and HNSCC patients adjusted by whether HPV infected tumor (lower panel). IL-6 *in vitro* secretion of purified monocytes determined by ELISA analyses following 24-hour LPS stimulation. Cytokine levels with background subtracted dichotomized by median value to low (continuous line) or high (hatched line) response. Cohort (upper panel): 55 HNSCC and 18 control patients. Cohort (lower panel): 48 HPV-negative and 7 HPV-positive HNSCC patients. Statistics by Kaplan-Meier analyses (Log rank test).

Among the HNSCC patients, the IL-6 secretion level following *in vitro* LPS stimulation of monocytes was found to predict the 15-year prognosis when analyzed by Kaplan-Meier (*P* = 0.009) (Fig 2). High values of IL-6 secretion predicted a low survival. This was valid, both with stimulated values directly analyzed and analyzed with stimulated values in which background levels were subtracted. When studied adjusted by age, gender, TNM stage and HPV status, the IL-6 secretion still predicted the 15-year prognosis (RR = 2.32; CI 1.18–4.55; *P* = 0.015) (Table 4). Among the HNSCC patients, Kaplan-Meier analyses showed that the LPS-stimulated IL-6 secretion rate predicted the 15-year survival when stratified by HPV status (*P* = 0.019), and only including the HPV-negative patients (*P* = 0.013) (Fig 2).

**Table 4:** HNSCC patient Cox regression 15-year overall survival analyses dependent on monocyte, lymphocyte and combined monocyte/lymphocyte function.

	Univariate			
	95% CI for HR			
	HR	Lower	Upper	Sig.
IL-6*	2.31	1.20	4.41	.012
CD71/CD3*	2.05	1.10	3.81	.023
Sum score**	2.15	1.30	3.56	.003
	Bivariate (HPV-adjusted)			
	95% CI for HR			
	HR	Lower	Upper	Sig.
IL-6*	2.07	1.07	3.98	.030
CD71/CD3*	2.04	1.10	3.78	.024
Sum score**	2.01	1.21	3.33	.007
	Multivariate (Age, gender, TNM stage, and HPV)			
	95% CI for HR			
	HR	Lower	Upper	Sig.
IL-6*	2.32	1.18	4.55	.015
CD71/CD3*	2.12	1.13	3.96	.019
Sum score**	3.11	1.68	5.75	.000

HR: Hazard ratio.

CI: Confidence interval.

* = binomially scored

** = Sum score: IL-6 + CD71/CD3 (both binomially scored)

### Prognostic value of PB lymphocyte activation status

**Fig 3:**
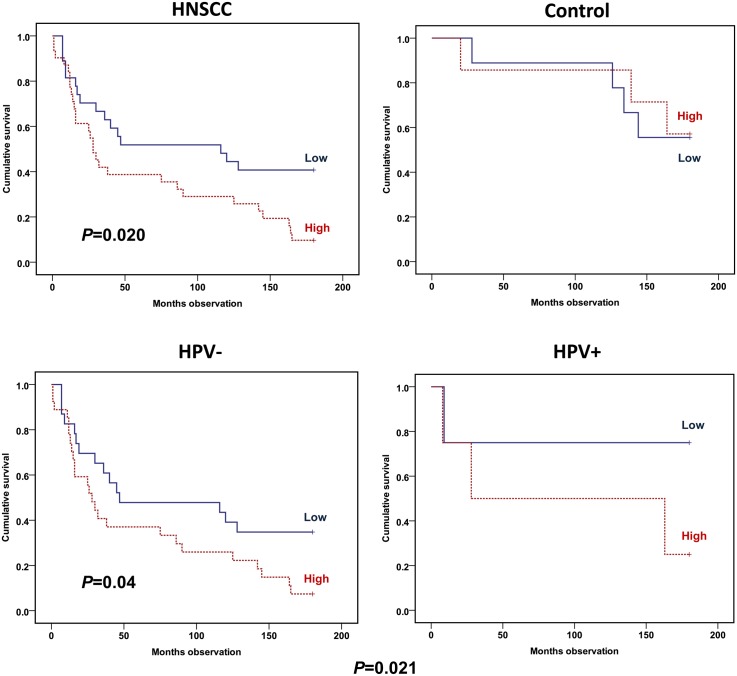
Overall survival plots dependent on percentage CD71 expression on T lymphocytes (CD3-positive cells). HNSCC versus control patients (upper panel) and HNSCC patients adjusted by whether HPV infected the tumor (lower panel). Percentages determined by flow cytometry on fresh PBMCs harvested from the patients. Percentages dichotomized by median value to low (continuous line) or high (hatched line). Cohort (upper panel): 58 HNSCC and 16 control patients. Cohort (lower panel): 50 HPV-negative and 8 HPV-positive. Statistics by Kaplan-Meier analyses (Log rank test).

The PB lymphocyte activation was investigated by flow cytometry by the determination of percentage of T cells exposing the activation epitope CD71 on the surface. When gated on lymphocytes, a high CD71 level predicted an adverse HNSCC patient 15-year survival (*P* = 0.020) (Fig 3). No such prediction was determined among the controls (Fig 3). When stratified by HPV status, a 15-year survival prediction was still obtained among the HPV-negative patients (*P* = 0.04) (Fig 3). An adjustment for age, gender and TNM stage, as well as HPV status, did not remove the prediction of the CD71 T lymphocyte-positive percentage for prognosis (RR = 2.12; CI 1.13–3.96; *P* = 0.019) (Table 3).

### Prognostic value of combined PB lymphocyte and monocyte activation status

**Fig 4:**
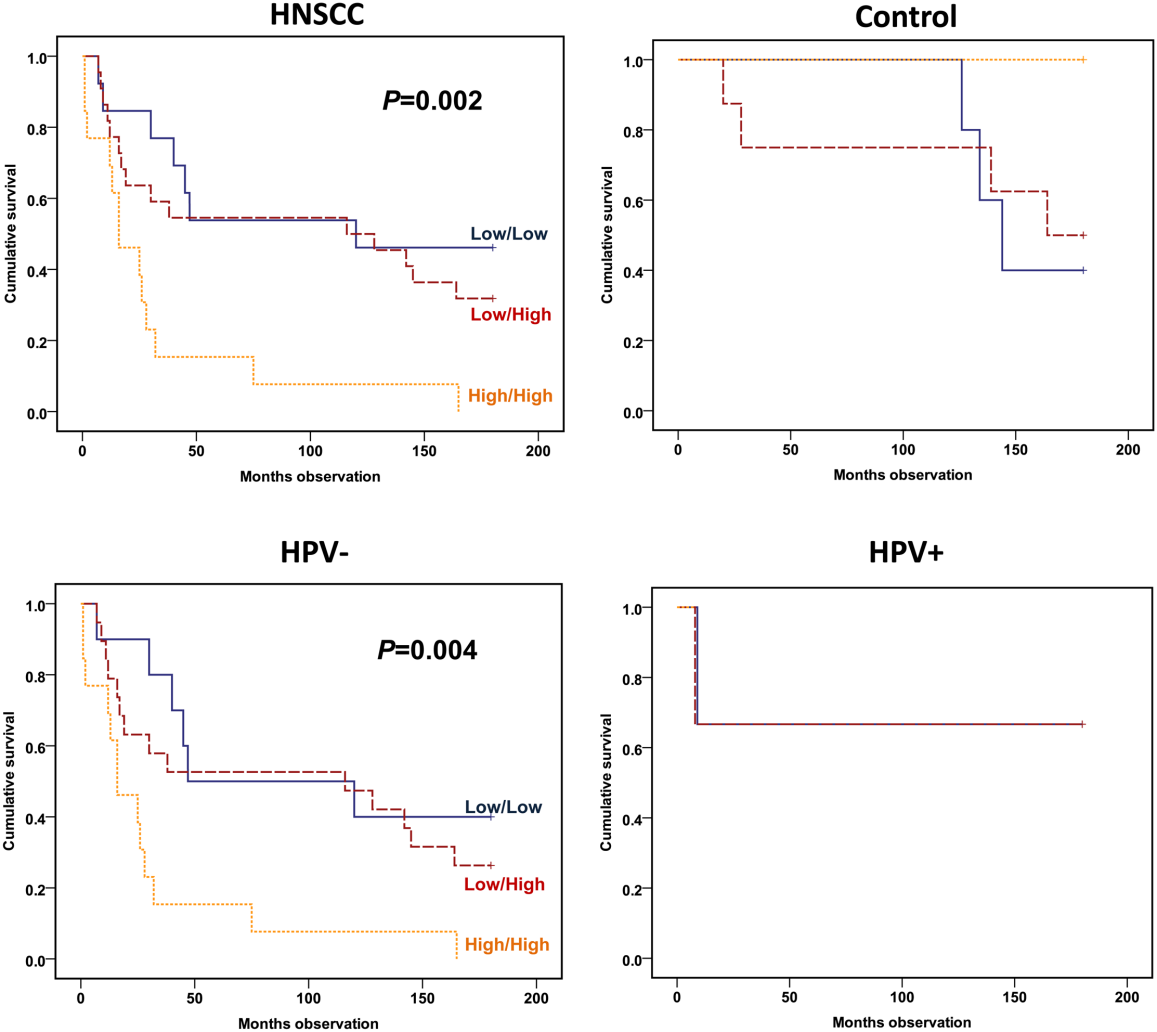
Overall survival according to low/high IL-6 monocyte secretion added to low/high CD71 T lymphocyte expression. HNSCC/control patients (upper panel) and HNSCC patients with HPV adjustment (lower panel). Monocytes cultured as in Fig 2 and lymphocytes analyzed as in Fig 3. Patients allocated to low IL-6 and CD71 (blue continuous line), low IL-6 or CD71 (red semi-hatched line) or both high IL-6 and CD71 (orange hatched line). Cohort (upper panel): 48 HNSCC and 16 control patients. Cohort (lower panel): 42 HPV-negative and 6 HPV-positive HNSCC patients. Statistics by Kaplan-Meier analyses (Log rank test).

If the CD71 T lymphocyte activation and IL-6 monocyte secretion, both dichotomized, were analyzed simultaneously using a Cox regression analysis and adjusted by age, gender, TNM stage and HPV status, both parameters had a significant prognostic impact on survival; i.e. IL-6 with RR = 3.25 (CI: 1.49–7.08; *P* = 0.003) and CD71/CD3 with RR = 2.99 (CI: 1.39–6.42; *P* = 0.005) (Table 5). Furthermore, these marker binomial scores were combined to one variable as a sum score. When analyzing the 15-year survival by including this variable, a highly significant negative prediction of the 15-year survival was obtained among the HNSCC patients (*P* = 0.002) (Fig 4). If stratified by HPV, a significant survival prediction was still obtained (*P* = 0.004) (Fig 4). With adjustments for age, gender, TNM stage and the HPV status of the patient, the combined activation variable still predicted survival (RR = 3.11, CI: 1.68–5.75; *P* = 0.000) when analysed by a Cox regression survival analysis (Table 4).

**Table 5:** HNSCC patient Cox regression survival analysis dependent on overall 15-year survival according to monocyte *in vitro* LPS-stimulated secretion of IL-6 and CD71 percentage expression on T lymphocytes adjusted by age, gender, TNM stage and whether HPV tumor infection.

	HR	95% CI for HR		
		Lower	Upper	Sig.
Age	1.05	1.01	1.10	.029
Gender	3.59	0.88	14.65	.075
T stage	2.19	1.48	3.26	.000
N stage	1.57	1.09	2.25	.015
HPV (±)	0.81	0.17	3.95	.792
IL-6*	3.25	1.49	7.08	.003
CD71/CD3*	2.99	1.39	6.42	.005

HR: Hazard ratio.

CI: Confidence interval.

* = binomially scored

**Fig 5:**
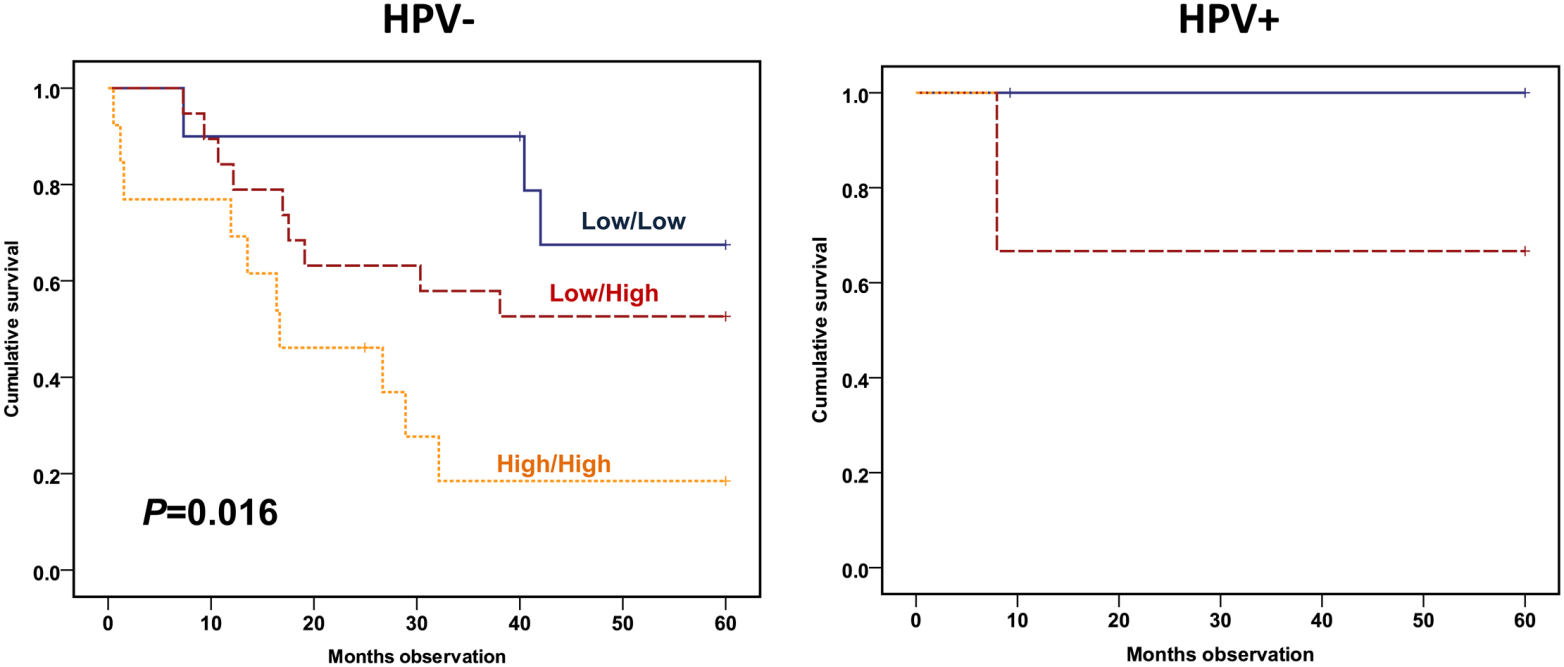
HPV adjusted disease-specific survival plot according to low/high IL-6 secretion added to low/high CD71 expression. Monocytes cultured as in Fig 2 and lymphocytes analyzed as in Fig 3. Patients allocated to low IL-6 and CD71 (blue continuous line), low IL-6 or CD71 (red semi-hatched line) or both high IL-6 and CD71 (orange hatched line). Cohort: 42 HPV-negative and 6 HPV-positive HNSCC patients. Statistics by Kaplan-Meier analysis (Log rank test).

If studied by disease-specific survival (DSS), the combined variable predicted DSS among the HPV-negative patients (*P* = 0.016) (Fig 5). When including all HNSCC patients available adjusted by age, gender, TNM stage and HPV status, the sum score still predicted DSS (RR = 5.55, CI: 2.31–13.37; *P* = 0.000) (Table 6).

**Table 6:** HNSCC patient Cox regression survival analysis dependent on disease-specific survival according to monocyte *in vitro* LPS-stimulated secretion of IL-6 and CD71 percentage expression on T lymphocytes sum score adjusted by age, gender, TNM stage, and whether there was HPV tumor infection.

	HR	95% CI for HR		
		Lower	Upper	Sig.
Age	1.07	1.01	1.13	.031
Gender	3.92	0.69	22.38	.125
T stage	3.23	1.84	5.69	.000
N stage	2.43	1.45	4.05	.001
HPV (±)	2.90	0.27	30.86	.379
Sum score*	5.55	2.31	13.37	.000

HR: Hazard ratio.

CI: Confidence interval.

* = Sum score: IL-6 + CD71/CD3 (both binomially scored)

## Discussion

In this study we have examined monocyte responsiveness, as well as lymphocyte activation with cells from PB from controls and HNSCC patients. We have investigated monocyte responsiveness as measured by *in vitro* LPS-stimulated responses in monocytes (IL-6 secretion), and lymphocyte activation by the presence of an early activation epitope on the surface of PB T lymphocytes (CD71). Patients with high monocyte responsiveness had a lower 15-year survival than patients with a low such response. Likewise, a high PB T lymphocyte activation state as measured by the CD71 percentage on the surface, predicted decreased 15-year survival. The measured monocyte and lymphocyte factors independently predicted prognosis and the data were also stratified by HPV tumor infection status without any change in the results.

Many different carcinoma diseases rising from the aero-digestive tract are suggested to be at least partially dependent on inflammation-derived signals [3], e.g. via cytokine secretion [22]. This applies to both lung [2] and gastro-intestinal cancer [23].

Monocytes are members of the MNP system [4], which secrete such cytokines in particular following activation [24, 25]. Monocyte function may be assessed by measuring cytokine secretion after *in vitro* stimulation of the TLR-4 receptor [26]. Such stimulation promotes the secretion in particular of pro-inflammatory interleukins (IL-1β, IL-6, TNF-α) [25], and modulates chemokine (MIP-1α/β, MCP-1) secretion [24].

IL-6 is a pluripotent cytokine with mostly stimulatory functions. IL-6 may, e.g. act as an autocrine or paracrine tumor growth factor [16], but also as an anti-apoptotic agent [27] on cancer cells, as is suggested to be the case in oral cavity cancer [28]. We have previously shown that high levels of IL-6 secreted from PB monocytes stimulated *in vitro* through the TLR-4 receptor herald a decreased intermediate-term survival [19]. In this updated study, a high IL-6 secretion still predicted survival following 15 years of observation. We have previously shown that monocytes are stimulated to secrete IL-6 upon HNSCC tumor co-culture [4]. The strength of this stimulation also yielded prognostic information [29], and the results suggest that IL-6 reflect an importance of the degree of inflammation upon HNSCC disease.

The measurement of the *in vivo* exposure of activation surface molecules on PB lymphocytes from HNSCC patients may be used as a marker for a general *in vivo* activation of the adaptive immune system [30]. We have previously shown that an increased percentage of these activation-related epitopes on T lymphocytes predicted a decreased survival [31]. This updated long-term survival study shows that a high CD71 percentage of positive T lymphocytes predicted a decreased survival. This is further in accordance with the results of Starska [32] and Millrud [33].

We have presently shown that the monocyte function was dependent on HPV status. It has been suggested that the immune system participates in the treatment-related clearance of HNSCC, particularly with HPV+ tumors [8]. A future aim would therefore be to study the immune system and tumor interaction more thoroughly and throughout the treatment of HPV+ versus HPV- HNSCC patients.

HPV+ HNSCC patients have a better prognosis by responding better to treatment than patients with HPV-negative tumors [34]. By stratifying survival analyses with HPV status, we have shown that both the shown lymphocyte and monocyte prognostic values were valid irrespective of HPV infection status. The findings support that the degree of inflammation is an important factor as to HNSCC prognosis, especially in HPV-negative patients, because the number of patients with HPV infection was currently too low to draw any firm conclusions.

IL-6 has been shown to be an inflammation-related growth promoter in many different cancer growth processes [16], but also in processes such as atherosclerosis and autoimmune disease [16]. Hence, it is conceivable that the shown IL-6 prediction also reflects a wider co-morbidity panorama than the HNSCC disease only. This should be investigated.

Measured monocyte activation responses, as well as the lymphocyte activation level, reflected the prognosis also when adjusted for TNM stage. Thus, the level of disease at diagnosis did not affect the present prognostic findings.

The monocyte and lymphocyte prognostic power has been shown to be independent of each other in the current investigation. This could be interpreted that the natural immune system, i.e. the MNP system, and the adaptive immune system are independently affected by the cancer disease. We have previously shown the same to be the case with stimulated monocyte MCP-1 secretion combined with T lymphocyte CD69 expression [13]. For this reason, several individual lines of measurement show similar results. To the best of the authors’ knowledge, this is among the first studied cohort in which unique survival predictions associated with PB natural and adaptive immune systems have been shown to independently predict prognosis in HNSCC patients. An especially interesting finding is that if the activation of both the natural and adaptive immune system were present, the prognosis was found to be particularly grim. This suggests a fatal consequence of combined lymphocyte activation and MNP priming.

It is generally held that virus-associated inflammation may be beneficial to the host [35], whereas inflammation caused by irritants and/or tumor cell-derived molecules have the opposite relation [36] to an established tumor. Our results suggest, at least in part, a more complex association than is currently often held. The implications of studying both general and local tumor immunity in cancer patients should be further studied.

Vaccination by the use of dendritic-pulsed cells has attracted a considerable amount of attention as a cancer treatment [37]. The fact that the immune system seems to be affected in HNSCC patients suggests that such therapy, specifically with HPV-related antigens, should be investigated further. Future studies may also explore the use of α-IL-6 antibodies and T lymphocyte inhibition as adjuvant treatments of the HNSCC disease.

## Conclusions

We have shown that monocyte function may be dependent on HPV infection status in HNSCC patients. Increased LPS-stimulated monocyte IL-6 secretion and a high activation of T lymphocytes uniquely predicted impaired HNSCC prognosis. The negative prognostic values were independent of TNM stages and HPV infection status. Thus, monocytes and lymphocytes seem to play an important role with respect to the clinical outcome in HNSCC patients.
